# Fibrinogen Interaction with Astrocyte ICAM-1 and PrP^C^ Results in the Generation of ROS and Neuronal Death

**DOI:** 10.3390/ijms22052391

**Published:** 2021-02-27

**Authors:** Nurul Sulimai, Jason Brown, David Lominadze

**Affiliations:** 1 Department of Surgery, University of South Florida Morsani College of Medicine, Tampa, FL 33612, USA; nurulsulimai@usf.edu (N.S.); jasonb3@usf.edu (J.B.); 2 Department of Molecular Pharmacology and Physiology, University of South Florida Morsani College of Medicine, Tampa, FL 33612, USA

**Keywords:** apoptosis, pro-inflammatory markers, neuron, NO, proximity ligation, ROS

## Abstract

Many neuroinflammatory diseases, like traumatic brain injury (TBI), are associated with an elevated level of fibrinogen and short-term memory (STM) impairment. We found that during TBI, extravasated fibrinogen deposited in vasculo-astrocyte interfaces, which was associated with neurodegeneration and STM reduction. The mechanisms of this fibrinogen-astrocyte interaction and its functional role in neurodegeneration are still unclear. Cultured mouse brain astrocytes were treated with fibrinogen in the presence or absence of function-blocking antibody or peptide against its astrocyte receptors intercellular adhesion molecule-1 (ICAM-1) or cellular prion protein (PrP^C^), respectively. Fibrinogen interactions with astrocytic ICAM-1 and PrP^C^ were characterized. The expression of pro-inflammatory markers, generations of reactive oxygen species (ROS) and nitric oxide (NO) in astrocytes, and neuronal death caused by astrocyte-conditioned medium were assessed. Data showed a strong association between fibrinogen and astrocytic ICAM-1 or PrP^C^, overexpression of pro-inflammatory cytokines and overproduction of ROS and NO, resulting in neuronal apoptosis and death. These effects were reduced by blocking the function of astrocytic ICAM-1 and PrP^C^, suggesting that fibrinogen association with its astrocytic receptors induce the release of pro-inflammatory cytokines, resulting in oxidative stress, and ultimately neuronal death. This can be a mechanism of neurodegeneration and the resultant STM reduction seen during TBI.

## 1. Introduction

Fibrinogen (Fg) is a soluble glycoprotein that is widely known for its role in the coagulation cascade of hemostasis. It is a high molecular weight (~340 kD) protein typically with a tri-nodular structure of about 46 nm in length [1], while its Stokes-Einstein radius is about 8.4 nm [2], which is significantly greater than that of, for example, albumin (~3.5 nm). Therefore, during normal physiological conditions, at blood content of ~2 mg/mL [3], Fg stays in the circulation, being easily kept away from the brain parenchyma by the blood-brain barrier (BBB) [3,4]. However, during pathology that is associated with an inflammation-induced compromised BBB, such as mild to moderate traumatic brain injury (TBI), Fg gains access to the extravascular space of the brain through extravasation, mainly via caveolar transcytosis [5]. In a clinical study, it was shown that, during TBI, Fg levels increased to 4 mg/mL and above for two days after injury and can remain high for as long as 14 days. [6]. Depositions of Fg and its derivative, fibrin, have been found in the brains of patients with neuroinflammatory diseases such as TBI [7,8], Alzheimer’s disease (AD) [9], and multiple sclerosis [10]. We found that during mild-to-moderate TBI, Fg crossed the vascular wall and deposited in the vasculo-astrocyte interface, where it was immobilized [5]. This is the location where Fg comes into close proximity with its possible receptors on the surface of astrocytes.

Intercellular adhesion molecule-1 (ICAM-1) is a transmembrane glycoprotein that is expressed in glial cells [11] and it is a well-known Fg receptor [12]. Another protein of interest is the cellular prion protein (PrP^C^) that is a cell surface, glycosylphosphatidylinositol-anchored glycoprotein that is abundantly expressed in nervous system cells such as neurons and glial cells [13]. PrP^C^ has been implicated in memory reduction [14,15]. While we have found that Fg can interact with astrocytic PrP^C^ [16], no direct visual evidence of this association was provided. Therefore, we aimed to investigate if Fg can associate with ICAM-1 and PrP^C^ on the surface of astrocytes using proximity ligation assay (PLA) and define if these interactions cause activation of astrocytic pro-inflammatory markers resulting in neuronal apoptosis and death.

Activation of astrocytic ICAM-1 and PrP^C^ through a possible interaction with Fg can lead to a series of effects [17]. We have previously shown that Fg interacts with ICAM-1 on endothelial cell surfaces causing vasoconstriction [18] and an increase in vascular permeability [19,20]. We have also shown that elevated levels of Fg, called hyperfibrinogenemia (HFg), causes astrocyte activation in vitro [21]. Whereas in an in vivo study, using the cortical contusion injury model of mild-to-moderate TBI, the deposition of Fg in the extravascular space resulted in a significant change of astrocyte morphology, indicating their activation that coincided with increase in neuronal degeneration [5]. However, the direct effect of HFg-induced astrocyte activation on neurons is not known.

Involvement of fibrin(ogen) in the generation of reactive oxygen species (ROS) in microglia and as a causative agent of oxidative damage has been well documented [22,23,24]. However, to the best of the author’s knowledge, Fg-induced temporal production of ROS in astrocytes has not been characterized. Therefore, in this study we aimed to investigate the effect of HFg on astrocyte activation and its effect on neuronal viability using an astrocyte-neuron co-culture model.

## 2. Results

### 2.1. Fibrinogen Interaction with Astrocytic ICAM-1 and PrP^C^

PLA is used to visualize ligand-receptor interactions at a single molecule resolution. We used this method to observe interactions between Fg and its receptors ICAM-1 and PrP^C^ on the surface of astrocytes. In a preliminary study, the use of a higher content of Fg (2 or 4 mg/mL), similar to those used in our previous studies [16,21], resulted in signal saturation. Therefore, we used lower doses of Fg to detect if there was an association of Fg with its astrocyte receptors. We have previously showed that astrocytes express ICAM-1 [21]. To specifically validate these results, we tested the expression of ICAM-1 in astrocytes in response to an interaction with Fg. Data presented in Figure S1 showed that Fg dose-dependently increased expression of ICAM-1 on the surface of astrocytes. These results coincide with data indicating that about 80% of rat astrocytes express ICAM-1 and that its expression increases with the activation of cells with an inflammatory cytokine [25]. Our data suggest that mouse astrocytic ICAM-1 is constitutively expressed in cells, but is overexpressed after the cell activation [26], in our case with Fg. Moreover, Fg dose-dependently increased astrocyte death (Figure S2).

**Figure 1:**
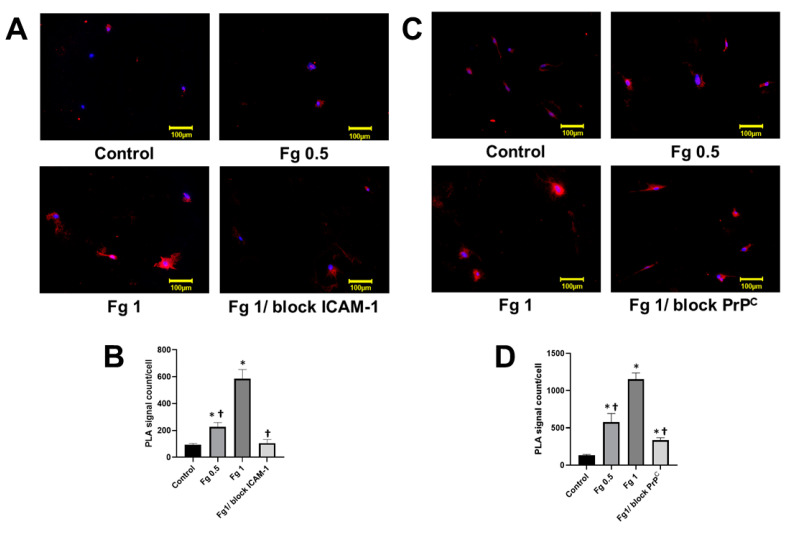
Interaction of fibrinogen (Fg) with its astrocytic receptors, intercellular adhesion molecule 1 (ICAM-1) and cellular prion protein (PrP^C^) detected by proximity ligation assay (PLA). (**A**) Representative images of in situ PLA signal (shown in red) depicting an interaction between Fg and astrocyte ICAM-1. Cells were treated with medium alone (control), 0.5 mg/mL of Fg (Fg 0.5), 1 mg/mL of Fg (Fg 1), and 1 mg/mL of Fg in the presence of function-blocking antibody against ICAM-1 (Fg1/block ICAM-1) for 17 h. The blocker of ICAM-1 function was added to cells 2 h prior to Fg treatment. Fg and ICAM-1 were detected using anti-Fg and anti-ICAM-1 antibodies, respectively. Cellular nuclei were labeled with 4′,6-diamidino-2-phenylindole (DAPI, blue). (**B**) PLA signals for Fg and ICAM-1 interactions were quantified and normalized per number of cells in each an image. (**C**) Representative images of in situ PLA signal (shown in red) depicting an interaction between Fg and astrocyte PrP^C^. Cells were treated with medium alone (control), 0.5 mg/mL of Fg (Fg 0.5), 1 mg/mL of Fg (Fg 1), and 1 mg/mL of Fg in the presence of PrP^C^ function-blocking peptide (Fg1/block PrP^C^) for 17 h. The blocker of PrP^C^ function was added to cells 2 h prior to Fg treatment. Fg and PrP^C^ were detected using anti-Fg and anti-PrP^C^ antibodies, respectively. Cellular nuclei were labeled with DAPI (blue). (**D**) PLA signals for Fg and PrP^C^ interactions were quantified and normalized per number of cells in each image. *p* < 0.05 in all; *—vs. Control, †—vs. Fg1; *n* = 4.

Minimal PLA positive signals were observed on surfaces of astrocytes that were not treated with Fg (Figure 1). In contrast to this, significantly more PLA positive signals were found on astrocytes treated with 0.5 mg/mL or 1 mg/mL of Fg showing a Fg dose-dependent increase of Fg-ICAM-1 and Fg-PrP^C^ interactions (Figure 1A–D). Astrocytes that were pre-treated with a function-blocking antibody against ICAM-1 and then treated with 1 mg/mL of Fg demonstrated a significantly attenuated PLA signal compared to the astrocytes treated with 1 mg/mL of Fg alone (Figure 1A,B). Similarly, treatment with a function-blocking PrP^C^ peptide reduced the PLA signal in astrocytes treated with Fg compared to those treated with Fg alone (Figure 1 C,D). However, the ratio of PLA signals in the astrocytes treated with 1 mg/mL of Fg in the presence of the ICAM-1 function blocker to that in astrocytes treated with 1 mg/mL of Fg (0.84 ± 0.02 PLA signal/cell) was greater than the ratio of PLA signals in the astrocytes treated with 1 mg/mL of Fg in the presence of PrP^C^ blocker to that in astrocytes treated with 1 mg/mL of Fg (0.70 ± 0.006 PLA signal/cell). For the results of the negative controls used in the study, please see Figure S3. The increased intensity in the PLA signal is indicative of an interaction between Fg and its astrocytic receptors. To confirm the specificity of the antibodies used and to validate our method, we observed the generation of PLA signals on astrocytes after systematically omitting each of the primary antibodies in a separate series of experiments (Figure S3). In addition, we tested the interaction of Fg and IgG that was used as a control for detecting antibodies against ICAM-1 and PrP^C^. Results showed no visible PLA signal in any of those experimental groups, confirming the validity of our experiments (Figure S3).

### 2.2. Fibrinogen-Induced Upregulation of Pro-Inflammatory Cytokines in Astrocytes

**Figure 2:**
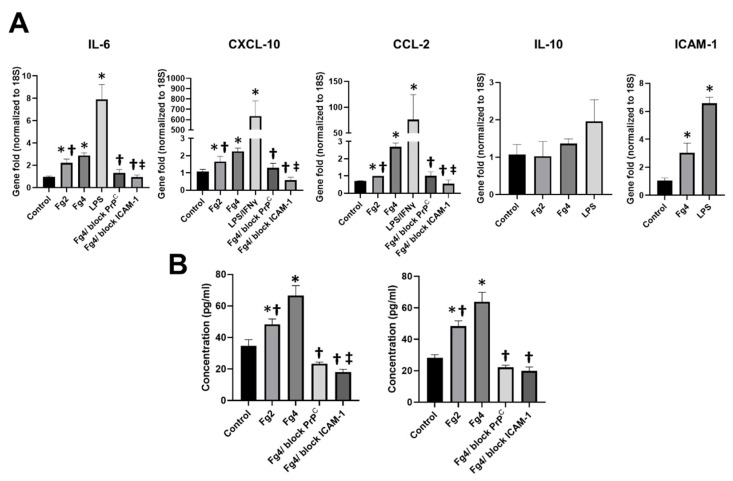
Fibrinogen (Fg)-induced expression of pro-inflammatory cytokines in astrocytes. (**A**) Gene expression of astrocyte pro-inflammatory cytokines interleukin 6 (IL-6), C-X-C motif chemokine 10 (CXCL10), C-C motif chemokine 2 (CCL2), intercellular adhesion molecule-1 (ICAM-1) and anti-inflammatory cytokine interleukin 10 (IL-10) in response to treatment overnight (17 hr) were detected with quantitative real-time polymerase chain reaction (qRT-PCR) analysis. Cells were treated with medium alone (control), 2 mg/mL or 4 mg/mL of Fg (Fg4), 4 mg/mL of Fg in the presence of PrP^C^ function-blocking peptide (Fg4/block PrP^C^) and 4 mg/mL of Fg in the presence of function-blocking antibody against ICAM-1 (Fg4/block ICAM-1). Lipopolysaccharide (LPS, 1 µg/mL), with or without co-stimulation with 20 ng/mL of a murine interferon gamma (IFNγ), was used as a positive control. Data were presented as a gene fold normalized to 18S, a housekeeping gene. (**B**) Content of the astrocytic IL-6 and CXCL-10 proteins in astrocyte conditioned media was measured by enzyme-linked immunosorbent assay. Cells were treated with medium alone (control), 2 mg/mL or 4 mg/mL of Fg, 4 mg/mL of Fg in the presence of a PrP^C^ function-blocking peptide (Fg4/block PrP^C^), and 4 mg/mL of Fg in the presence of a function-blocking antibody against ICAM-1 (Fg4/block ICAM-1). *p* < 0.05 in all; *—vs. Control, †—vs. Fg4 and ‡—vs. Fg4/block PrP^C^; *n* = 6.

Fg dose-dependently increased gene expression of pro-inflammatory cytokines interleukin 6 (IL-6), C-X-C motif chemokine 10 (CXCL-10), and C-C motif chemokine 2 (CCL2) in astrocytes (Figure 2A). At high concentration, Fg also upregulated ICAM-1 gene expression on the surface of astrocytes. However, Fg effects were not as strong as the effects induced by lipopolysaccharide (LPS), with or without co-stimulation with murine interferon gamma (IFNγ), which was used as a positive control (Figure 2A). Fg did not affect gene expression of anti-inflammatory cytokine interleukin 10 (IL-10) in astrocytes. The increase seen in IL-6, CXCL-10 and CCL-2 gene expressions induced by HFg was ameliorated when astrocytes were treated with function-blocking ICAM-1 antibody or PrP^C^ function-blocking peptide (Figure 2A).

Similar results were found with IL-6 and CXCL-10 protein expressions detected by enzyme-linked immunosorbent assay (ELISA) (Figure 2B). There was a dose-dependent increase of IL-6 and CXCL-10 protein contents in media from HFg-treated astrocytes (Figure 2B). The presence of a function-blocking ICAM-1 antibody or PrP^C^ function-blocking peptide significantly decreased the content of IL-6 and CXCL-10 proteins in the media taken from astrocytes treated with a high concentration (4 mg/mL) of Fg (Figure 2B). Blocking of the astrocytic ICAM-1 function more effectively decreased the expression of IL-6 than blocking the function of PrP^C^ (Figure 2B). The blocking of Fg astrocytic receptors ICAM-1 and PrP^C^ ameliorated Fg-induced expression of CXCL-10 in astrocytes to almost a similar extent (Figure 2B).

### 2.3. Fg-Induced Generation of ROS in Astrocytes

**Figure 3:**
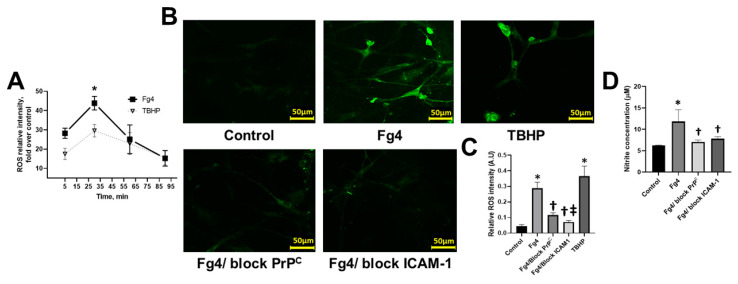
Fibrinogen (Fg)-induced generation of reactive oxygen species (ROS) and the production of nitric oxide (NO) in astrocytes. (**A**) The kinetics of astrocyte ROS formation induced by Fg was measured by luminol-enhanced chemiluminescence assay. Astrocytes were treated with 4 mg/mL of Fg (Fg4) or tert-butyl hydroperoxide (TBHP) used as a positive control. A two-way ANOVA test indicated that the effect of time and treatment on ROS production was significant with *p* < 0.0001 for both factors. *p* < 0.05; *—vs. time and treatment group; *n* = 4. (**B**) Representative images show ROS generation by astrocytes in response to treatment with medium alone (control), 4 mg/mL of Fg (Fg4), and 4 mg/mL of Fg in the presence of a function-blocking peptide against cellular prion protein (Fg4/block PrP^C^) or 4 mg/mL of Fg in the presence of function-blocking antibody against intercellular adhesion molecule-1 (Fg4/block ICAM-1). TBHP was used as a positive control. (**C**) Summary of image analyses for the detection of Fg-induced ROS generation in astrocytes. *p* < 0.05 in all; *—vs. Control, †—vs. Fg-4 and ‡—vs. Fg4/block PrP^C^; *n* = 6. (**D**) Production of astrocyte NO in response to Fg treatment was assessed by measuring NO content in astrocyte media after experiments using a Griess reaction assay. Cells were treated with medium alone (control), 4 mg of Fg, 4 mg/mL of Fg in the presence of a function-blocking peptide against PrP^C^ (Fg4/block PrP^C^), and 4 mg of Fg in the presence of a function-blocking antibody against ICAM-1 (Fg4/block ICAM-1). *p* < 0.05 in all; *—vs. Control, *n* = 4.

We examined the kinetics of Fg-induced generation of ROS in astrocytes. It showed that ROS production was increased in the first 30 min followed by a slow reduction during the next 1 h (Figure 3A). However, it remained greater than that in the control group at all time points (Figure 3A).

Astrocytes that were treated with Fg generated more than double the amount of ROS than those in the control group (Figure 3B,C). When astrocytic ICAM-1 or PrP^C^ functions were blocked, the Fg-induced ROS generation was significantly lowered compared to that in astrocytes treated with Fg alone (Figure 3B). However, blocking the function of ICAM-1 resulted in a greater reduction of ROS generation than that caused by blocking the function of PrP^C^.

The level of nitric oxide (NO) was increased in the astrocyte-conditioned medium from astrocytes treated with 4 mg/mL of Fg. Use of a function-blocking antibody against ICAM-1 or PrP^C^ function-blocking peptide significantly lowered the Fg-induced NO production (Figure 3D).

### 2.4. Fg-Activated Astrocytes Increased Neuronal Apoptosis and Death

**Figure 4:**
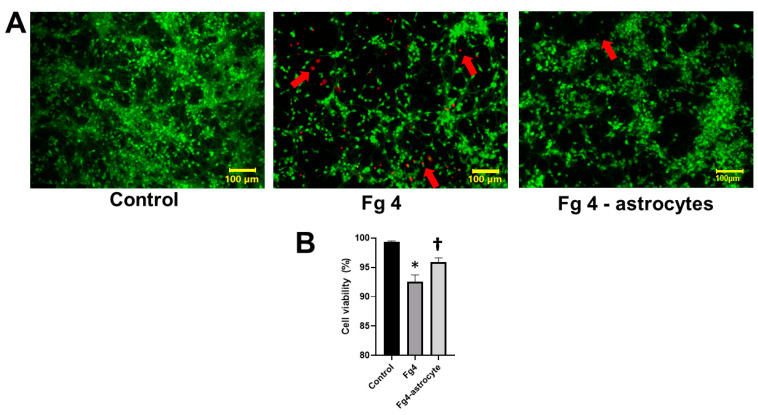
Fibrinogen (Fg)-induced astrocyte activation increased neuronal cell death. (**A**) Representative images show live/dead staining of neurons co-cultured with astrocytes that were treated with 4 mg of Fg (Fg4) or neurons that were left growing on the bottom of the well with 4 mg/mL of Fg on the Transwell^®^ membrane with no astrocytes (Fg4-astrocytes) for 24 h. Live neurons were detected by uptake and trapping of calcein AM (green fluorescence) while dead cells were the ones that were unable to trap calcein AM and were permeable to ethidium homodimer (red fluorescence). Red arrows indicate dead neurons. (**B**) Summary of image analyses for the detection of live neurons presented as a percent of a total number of neurons. An automatic cell count was performed, based on the fluorescence signal threshold of images provided by the CellSens Dimension software. Well-defined “segments” were counted to define number of dead cells as a percent of a total number of cells in the same image. Data were averaged for each group. *p* < 0.05; *—vs. Control, †—vs. Fg4; *n* = 6.

Neurons co-cultured with astrocytes that were treated with 4 mg/mL of Fg showed significantly higher death compared to that in the untreated control group (Figure 4). In addition, the direct effect of Fg (4 mg/mL) placed on Transwell^®^ membrane inserts without astrocytes was significantly lesser than that caused by Fg-activated astrocytes (Figure 4). The concentration of Fg in the bottom chambers of the Transwell^®^, measured by ELISA, was significantly lower (0.1 ± 0.04 mg/mL) than that (0.5 ± 0.07 mg/mL) in the upper chambers of the system after 24 h, while the initial level of Fg in the upper chamber of the Transwell^®^ was 4 mg/mL.

**Figure 5:**
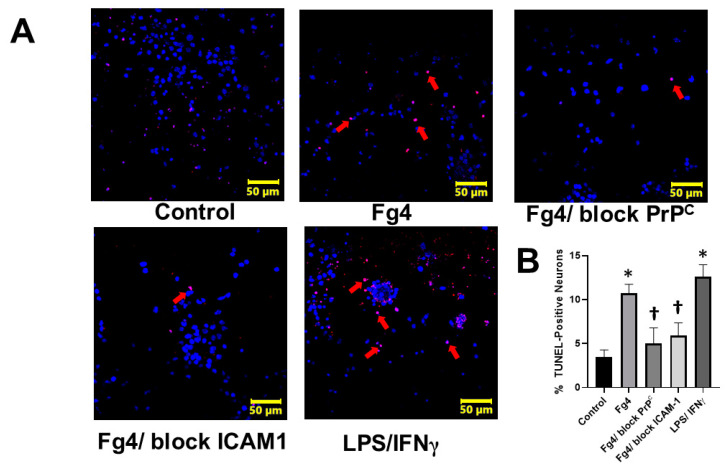
Fibrinogen (Fg)-induced astrocyte activation causes apoptosis of co-cultured neurons (**A**) Representative images of neuronal apoptosis assessed by Terminal deoxynucleotidyl transferase-dUTP nick end labeling (TUNEL) assay. TUNEL assay is a method for detecting DNA fragmentation by labeling the 3′- hydroxyl termini in the double-strand DNA breaks generated during apoptosis. Neurons were co-cultured with astrocytes treated with Fg for 24 h. Astrocytes were treated with medium alone (control), 4 mg/mL of Fg (Fg4), 4 mg/mL of Fg in the presence of a function-blocking peptide against cellular prion protein (Fg4/block PrP^C^) and 4 mg of Fg in the presence of function-blocking antibody against intercellular adhesion molecule-1 (Fg4/block ICAM-1). For positive control, lipopolysaccharide (LPS) was used at 1µg/mL with co-stimulation with 20 ng of a murine interferon gamma (IFNγ). Immunostaining of apoptotic (red) neurons with 4′,6-diamidino-2-phenylindole (DAPI, blue) nuclear stain is shown. Arrows indicate some of the apoptotic cells (red). (**B**) Summary of image analyses for the detection of apoptotic neurons presented as a percent of a total number of cells. *p* < 0.05 in all; *—vs. Control, †—vs. Fg4; *n* = 4.

This effect coincided with an increase in apoptosis caused by a high level of Fg, as determined by a terminal deoxynucleotidyl transferase-dUTP nick end labeling (TUNEL) assay (Figure 5). Inhibiting the interaction of Fg with astrocytic PrP^C^ or ICAM-1, using a function-blocking peptide or antibody, respectively, protected neurons from the cytotoxic effects of Fg-activated astrocytes (Figure 5).

## 3. Discussion

Many neurodegenerative diseases (e.g., TBI and AD) that are accompanied with memory reduction are typically associated with increased pro-inflammatory responses such as elevated levels of pro-inflammatory cytokines [4,27] and blood content of Fg [28]. TBI is a leading cause of neurological deficits in the brain, with the main associated problem being a decline in cognitive function and STM deficit [29]. Although the specific mechanism of TBI-induced cognitive impairment is not well understood, a strong association of TBI with inflammation is well recognized [30,31]. It has been shown that Fg and fibrin induce overexpression of pro-inflammatory cytokines in peripheral blood mononuclear cells [32] and microglia [23]. Therefore, it is possible that during TBI, when Fg extravasates from the blood vessels, it continues causing pro-inflammatory effects in the brain leading to secondary brain damage [5]. It has been shown that neuronal cell damage or the death of axons as well as cell bodies in the brain is a possible mechanism for the mild TBI-induced cognitive decline, and apoptosis is a key mechanism for secondary or delayed neuronal cell death during TBI [33,34]. Clinically, Fg immunoreactivity can be found in postmortem brain sections taken from patients anywhere from hours up to as long as 18 years after a fall [35]. Pro-Inflammatory effects of excess Fg, such as vasoconstriction, vascular permeability, and astrocyte, neuronal and glial activation, are well documented [18,21,22,36]. We have shown previously that deposition of extravasated Fg between a vessel and astrocyte endfeet in the animal model of TBI coincides with increased neurodegeneration and a reduction in STM [5]. However, besides causing physical detachment of vessels and astrocytes [5], molecular mechanism involved in extravasated Fg- and deposited Fg/fibrin-induced astrocyte-mediated neurodegeneration was not clear.

A growing body of evidence has pointed to astrocytes as key regulators of neuroinflammation [37]. Upon contact with certain stimuli or a ligand like e.g., Fg as a result of an inflammatory disease, such as TBI, activated astrocytes produce and secrete a variety of bioactive molecules that may influence the phenotype of the astrocytes and cause them to be detrimental to neurons and to the central nervous system as a whole [38].

The proinflammatory role of Fg/fibrin is manifested in its ability to bind to certain integrin receptors through a ligand-receptor interaction and activate a wide range of immune cells [22]. In this study, we aimed to investigate the interaction of the soluble protein Fg with its receptors on the surface of astrocytes, defining possible resultant inflammatory responses in astrocytes, which may result in their neurotoxicity. Use of hirudin in our experiments prevented the conversion of Fg into fibrin by thrombin. An interaction of Fg with ICAM-1 and PrP^C^ was tested using PLA, which allows highly specific and sensitive immunofluorescent detection to be visualized and objective quantification of protein-protein interactions in situ. The data showed that Fg was co-localized and thus strongly associated with astrocyte ICAM-1 and PrP^C^, suggesting that it is possibly binding to these receptors on the surface of astrocytes. This is the first time a Fg specific interaction with astrocytes was visualized, confirming our previous finding that Fg could be associated with ICAM-1 [21] and PrP^C^ with high specificity [16,36,39]. Thus, the increased respective PLA signals can be the result of a Fg interaction with over-expressed astrocyte ICAM-1 and PrP^C^.

Furthermore, Fg’s association to proteins such as PrP^C^ could lead to the formation of complexes that cause neurodegeneration [5,36,39]. PrP^C^ has been shown to have a dual effect in the brain: either neuroprotective or causing neuronal apoptosis and death when ligated [40,41]. Ligation of astrocyte PrP^C^ with Fg found in the present study, can be a mechanism of astrocyte-mediated neurotoxicity demonstrated here. 

The data showed that Fg increased IL-6 expression in astrocytes without affecting expression of anti-inflammatory cytokine IL-10. Clinically, increased IL-6 can be detected in serum and cerebrospinal fluid of human patients with TBI [42]. Thus, our data suggest that, during inflammation (i.e., TBI), an interaction of extravasated Fg with astrocytes may be involved in increased levels of IL-6 in serum and cerebrospinal fluid. It is known that the timing of the IL-6 peak increase in the serum correlates with an increase of acute-phase proteins, including Fg [42]. In addition, a Fg-induced increase in astrocyte IL-6 expression could potentially contribute to exacerbating pro-inflammatory conditions by triggering a positive feedback loop that enhances IL-6-induced Fg synthesis [43].

Our observations of Fg-induced increases in astrocytic IL-6, CXCL-10 and CCL2 along with previously shown overexpression of tyrosine receptor kinase B [21] and PrP gene [16], suggest that Fg induces activation of astrocytes with functional polarization towards their neurotoxic, A1 phenotype [44,45], potentially exacerbating inflammatory conditions typically found during TBI [46].

Data shows that the Fg-induced increases in inflammatory cytokines in astrocytes was ameliorated when treated with a function-blocking antibody to ICAM-1 or peptide to PrP^C^, indicating that the astrocyte pro-inflammatory response occurs in part via ligation of ICAM-1 and PrP^C^ and the subsequent signaling. Blocking PrP^C^’s function significantly reduced the PLA signals compared to the Fg1 group without the PrP^C^ function-blocker peptide. However, blocking ICAM-1′s function resulted in even greater PLA signal reduction from Fg1 group. Our data indicate that astrocytic ICAM-1 and PrP^C^ act synergistically (possibly additively in some cases) during the interaction between Fg and astrocytes. However, the significant difference in the effects of a Fg interaction with its astrocyte surface receptors ICAM-1 and PrP^C^ suggests that the Fg and astrocyte ICAM-1 interaction may have a more pronounced effect on astrocyte functional changes than the Fg/PrP^C^ interaction. ICAM-1 is considered a marker of inflammation and tissue damage [47,48]. Its expression can be upregulated in response to pro-inflammatory cytokines [47]. On the other hand, it has been shown that ligation of ICAM-1 on the surface of endothelial cells and astrocytes leads to the activation of pro-inflammatory signaling cascades [49] and expression of pro-inflammatory cytokines [50]. Previously we found that an interaction between Fg and astrocytes resulted in overexpression of the ICAM-1 protein [21]. Data of the present study indicate that Fg causes over expression of ICAM-1 and results in astrocytic ICAM-1 ligation. Combined, these results can be a mechanism of a Fg-astrocytic ICAM-1 interaction-induced increase in expression of pro-inflammatory cytokines. In these conditions, it is possible that the ICAM-1-induced expression of pro-inflammatory cytokines [50] exacerbates overexpression of ICAM-1 [47], leading to the formation of a positive feedback loop that results in an inflammatory condition, causing neuronal apoptosis and death.

An interaction with PrP^C^ evidently plays a critical role in mediating the synaptic deficit induced by soluble oligomers of amyloid-beta (Aβ) [17]. It has also been shown that Fg-Aβ and Fg-PrP^C^ complexes are associated with reduced cognitive function and STM in patients and mice, respectively [36,51]. Our findings confirm and strengthen the hypothesis that during TBI, extravasated Fg that deposits in vasculo-astrocyte interfaces interact with astrocytic ICAM-1 and PrP^C^, resulting in overexpression of PrP^C^ and the formation of a Fg-PrP^C^ complex, which could lead to alteration of astrocyte function, causing its activation and overexpression of pro-inflammatory cytokines. The reactive, A1 state of astrocytes is compounded by the oxidative stress produced by the increased production of ROS and NO that are induced by Fg as we have shown here. These effects are positively involved in neurodegeneration, leading to the memory reduction seen in vivo previously [5]. Our findings coincide with the work of others showing fibrin as an activator of the Nicotinamide adenine dinucleotide phosphate oxidase (NAPDH) complex that induces the release of ROS [23].

We found that an interaction between Fg and astrocytes caused an increase in the generation of ROS and production of NO. It is known that NO modulates and could increase the level of ROS in cells [52], which is known to cause neurodegeneration [40,53]. It has been shown that astrocyte PrP^C^ could be another source of ROS production via NAPDH and extracellular regulated kinases 1/2 signaling and may be involved in cell-redox homeostasis ROS [54]. This PrP^C^-induced ROS formation results in neuronal and other brain cell toxicity as well as oxidative stress [54,55]. Thus, our data showed that an interaction between Fg and its astrocyte receptors can be a triggering mechanism for the generation of NO and ROS by astrocytes.

Our data showed that Fg’s interaction to astrocytic ICAM-1 or PrP^C^ had, in general, a synergistic effect. In most of the cases, such as in PLA, IL-6, CXCL-10, and CCL-2 gene expressions, IL-6 protein expression, and ROS generation, the effects of Fg’s interaction with ICAM-1 was greater than those that resulted from Fg and PrP^C^ interactions. These results suggest that Fg may have a greater affinity with ICAM-1 than with PrP^C^. However, blocking the function of one of the Fg receptors or the other, in many cases, ameliorated the effect, indicating that there may be some freedom of choice when selecting the target to reduce Fg’s effects on astrocytes. However, the data indicate that ICAM-1 is likely the preferable target.

The TUNEL assay is a reliable test to label cells with fragmented DNA as a marker of apoptosis and has been widely validated in neurons [56]. Our data showed that the content of medium from Fg-activated astrocytes increased apoptosis in neurons as detected by a TUNEL assay. These results were supported by decreased viability in neurons co-cultured with astrocytes treated with Fg. Since these effects were ameliorated in the presence of the function-blocking antibody against ICAM-1 and the function blocking PrP^C^ peptide, it is apparent that Fg interacts with astrocyte receptors and the resultant activation of these astrocytes may cause the release of inflammatory cytokines and the generation of NO and ROS, which ultimately result in neuronal degeneration and their increased death.

The interaction between Fg and its receptors on astrocyte surfaces not only activates astrocytes leading to release of pro-inflammatory cytokines and the generation of both NO and ROS, but may be a mechanism for the conversion of astrocytes to phagocytic cells. It has been shown that astrocytes endocytose Fg/fibrin [57]. However, this act results in astrocyte death, shown in the same work (but not emphasized) [57] and in our previous study [5,21]. Experiments conducted specifically to address this matter showed that the interaction between Fg and astrocytes resulted in astrocyte death. Furthermore, we found that neuronal death was increased when co-cultured with Fg-activated astrocytes, suggesting a direct effect of activated astrocytes on neurons. This effect could be the result of the release and/or generation of toxic elements, such as inflammatory cytokines and ROS and NO, by activated astrocytes, which can lead to neuronal death. On the other hand, a direct interaction of Fg with neurons resulted in less neuronal death, suggesting a strong effect of activated astrocytes. A significantly low content of Fg in the bottom of Transwell^®^ chambers containing astrocytes grown in the inserts suggests that most of the Fg binds to astrocytes. In addition, some Fg can be endocytosed by activated astrocytes, as it is suggested elsewhere [57]. Thus, an interaction between Fg and astrocytes results in a cascade of effects that may lead to neuronal dysfunction associated with memory reduction during the TBI seen in our previous studies [5].

In conclusion, in the present study, we showed that Fg is positively associated with its receptors ICAM-1 and PrP^C^ on the surface of astrocytes. This association results in activation of astrocytes manifested by overexpression of pro-inflammatory cytokines and generation of NO and ROS. All of which, most likely, cause oxidative damage, leading to neuronal death and thus, the neurodegeneration seen during TBI. These effects can be a mechanism for the reduction of STM during TBI that is associated with extravasation of Fg and its deposition in vasculo-astrocyte interface as seen previously [5,36].

## 4. Materials and Methods

### 4.1. Antibodies and Reagents

Human Fg depleted of plasminogen, von-Willebrand factor, and fibronectin was from Enzyme Research Laboratories (South Bend, IN, USA). Polyclonal rabbit antibody against human Fg (cross-reacts with mouse) was purchased from Dako Cytomation (Carpentaria CA, USA). Rat purified function-blocking antibody (clone: YN1/1.7.4) against mouse ICAM-1 (CD-54, cat. # 116133) was obtained from BioLegend (San Diego, CA, USA) and prion protein (PrP^C^) blocking peptide (GTX89339-PEP) was from GeneTex (Irvine, CA, USA). For detection of ICAM-1 and PrP^C^, we used ICAM-1/CD54 antibody raised in mouse (cat. # NBP2-22541) from Novus biologicals (Littleton, CO, USA) and anti-prion protein antibody also raised in mouse (cat. # P0110) from Sigma Aldrich Chemicals Co (St. Louis, MO, USA), respectively. In vitro animal astrocyte medium (AM-a) was purchased from ScienCell Research Laboratories (Carlsbad, CA, USA). Poly-D-Lysine, laminin, hirudin, LPS from Escherichia coli (O111:B4), and Duolink^TM^ In Situ Detection Reagent Red were from Sigma. Recombinant murine interferon gamma (IFNγ) was purchased from Peprotech (Rocky Hill, NJ, USA).

### 4.2. Cell Culture

Mouse astrocytes from C57BL/6 were purchased from ScienCell. The astrocytes were grown in AM-a complete media in 8-well glass-bottomed plates from Millipore Sigma (Burlington, MA, USA) for PLA or on 12-well cell culture plate from Nunc™ (Thermo Fisher Scientific) to assess target gene and protein expressions with polymerase chain reaction (PCR) and ELISA, respectively. Poly-D-Lysine (30 µg/mL) and laminin (2 µg/mL) were used to coat plates for better astrocyte attachment. Cells were kept at 37 °C with 5% CO_2_ in a humidified environment as recommended by the manufacturer and were then used at the 3rd or 4th passages for the experiments. Primary mouse brain cortex neurons from C57BL/6 mice (cat. # M-cx-300) were purchased from Lonza and were grown in Primary Neural Basal Medium and PNGM^TM^ Single Quots^TM^, as recommended by the manufacturer. Twenty-four well plates, with or without #1 glass-bottomed coverslips, were used for seeding neurons and the Transwell^®^ inserts used for astrocyte growth were coated with Poly-D-Lysine and laminin.

**Figure 6:**
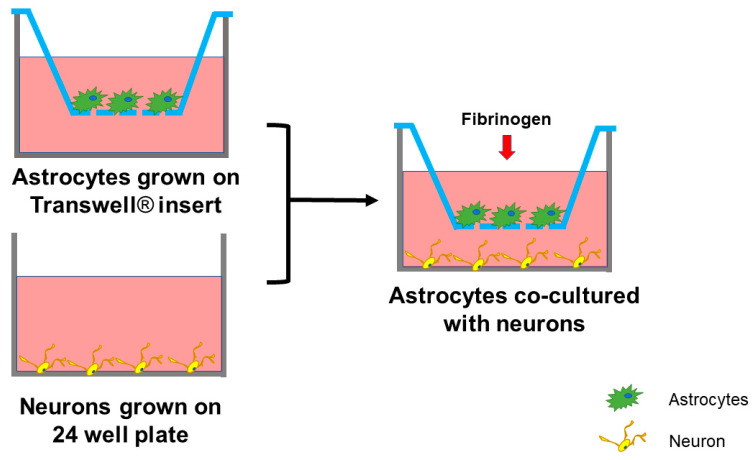
Co-culture of astrocytes and neurons. Neurons were cultured separately in 24-well plates for at least 7 days. Astrocytes were seeded separately on the Transwell^®^ permeable inserts. Once they reached 80% confluency, the inserts with astrocytes were moved to the wells with cultured neurons. After treating of astrocytes with treatment compounds, the cells were co-cultured for 24 h.

For the astrocyte-neuron co-culture system used (Figure 6), neurons were cultured separately in a #1 glass-bottomed 24-well plates for at least 7 days, while astrocytes were seeded separately on the 6.5 mm diameter Transwell^®^ permeable support inserts, with 0.4 μm diameter pores size polycarbonate membranes from Corning (New York, NY, USA). Astrocytes were plated at 1 × 10^5^ cells per Transwell^®^ insert (Figure 6) and used when they reached 80% confluency. Neurons were seeded in wells at the same density of 200,000 cells/well.

To investigate the contribution of Fg-induced astrocyte-secreted factors on neuron survival, astrocytes were seeded on a Transwell^®^ permeable supports placed on the top of wells as a “feeder layer” with neurons being grown on the bottom of the wells (Figure 6). This allowed us to culture neurons with media where astrocyte-secreted factors were present without neurons being in direct contact with astrocytes (Figure 6).

### 4.3. Experimental Setups and Groups

The complete media was removed the day of the experimentation and replaced with serum free medium (SFM) for 2 h. For PLA, serum-starved cells were treated with SFM alone, 0.5 mg of Fg, 1 mg/mL of Fg, 1 mg/mL of Fg in the presence of 22 μL ICAM-1 function-blocking antibody or 20 μL of PrP^C^ function-blocking peptide in SFM. For cytokine studies, the astrocytes were treated as described above, but with higher doses of Fg (2 or 4 mg/mL). For positive control, LPS was used at 1 µg/mL with or without co-stimulation with 20 ng/mL of a murine IFNγ. Each experimental group contained hirudin (1 U/mL) to inhibit any possible effect of thrombin converting Fg into fibrin. The cells were kept in an incubator at 37 °C for 17 h.

### 4.4. Proximity Ligation Assay

Astrocytes were rinsed with phosphate-buffered saline (PBS) before being fixed with 4% paraformaldehyde in PBS for 15 min and permeabilized with 0.05% TritonX-100 for 10 min. PBS was used as a washing solution during the procedure. In situ PLA was performed on treated astrocytes following the protocol suggested by the manufacturer. Briefly, the cells were incubated with Duolink^®^ blocking solution overnight at 4 °C. Primary antibodies were prepared in the Duolink^®^ antibody diluent. Two sets of experiments were performed on astrocytes: one using PLA to probe with anti-Fg (1:400) and ICAM-1 (1:150) antibodies and another using antibodies against Fg (1:400) and PrP^C^ (1:100). The astrocytes were incubated with the antibody pairs for 1.5 h at 37 °C. Cells were washed twice with wash buffer prior to incubation with Duolink^®^ PLA probes for 1 h at 37 °C. The primary antibodies act as antigens to the PLA oligonucleotide-conjugated plus and minus probes. The plus and minus probes are secondary antibodies against the two species matching the host species of the primary antibodies (anti-mouse and anti-rabbit) conjugated with plus and minus Duolink^®^ PLA probes (1:5). Cells were washed twice with wash buffer prior to incubation with the Duolink^®^ ligation-ligase solution (30 min at 37 °C) followed by incubation with the amplification-polymerase solution (100 min at 37 °C). Then cells were washed with another wash buffer and mounted with the Duolink^®^ PLA mounting medium with 4′,6-diamidino-2-phenylindole (DAPI). PLA signals (red, λ_excitation/emission_ 598/634 nm) were identified as fluorescent spots with a fluorescence microscope whenever one molecule of Fg was within the proximity of less than 40 nm with its pair, ICAM-1 or PrP^C^.

PLA is a highly specific and sensitive method to detect protein-protein association [58]. Two primary antibodies raised in different species are typically used to detect two protein targets. A pair of secondary antibodies that has been labeled with oligonucleotide (PLA probes), bind to the primary antibodies. Next, the PLA probes are joined by connector oligos to become ligated, resulting in closed, circular DNA template. It is only possible for the connector oligos to join the pair of the PLA probes simultaneously when the two proteins are in close proximity with each other. The resultant circular DNA template later undergoes rolling-circle amplification, where the PLA probes act as a primer for a DNA polymerase, which generates copies of the sequences, amplifying the signal that is still anchored to the PLA probe and allowing detection of its localization. Complementary detection oligos coupled to fluorochromes hybridized to repeating sequences in the amplicons allow the individual interacting pairs of proteins to be visualized and counted. All the reagents and buffers used for the PLA were provided by the manufacturer in the kit.

### 4.5. Quantitative Real-Time PCR (qRT-PCR)

Total RNA was extracted from mouse astrocytes using TRIzol reagent (Invitrogen) and reverse transcription was conducted using an iScript cDNA synthesis kit from Bio-Rad (Hercules, CA, USA), following the manufacturer’s instruction. qRT-PCR analysis was carried out using PowerUp™ SYBR™ Green Master Mix (Applied Biosystems, Austin, TX, USA). The PCR cycle parameters were: 50 °C for 2 min, followed by 95 °C for 10 min, then 40 cycles at 95 °C for 15 s, and the annealing temperatures were kept between 56 to 60 °C for 1 min, according to optimized annealing temperature for the different set of primers. Gene expression levels were determined by QuantStudio 3 from Life Technologies (Carlsbad, CA, USA). The mRNA expression of target genes was analyzed and normalized to 18S, which was used as the housekeeping gene. Data analysis of fold changes in gene expression was perfomed using the ΔΔCt method and presented as 2^−(average ΔΔCt)^. The following primers were used: IL-6-Fwd 5’-GACTTCCATCGAGTTGCCTTCT-3′, Rev 5′-TTGGGAGTGGTATCCTCTGTGA-3′; CXCL-10-Fwd 5′-AAGTGCTGCCGTCATTTTCT-3′, Rev 5′-GTGGCAATGATCTCAACACG-3′; CCL2-Fwd 5′-GTTGGCTCAGCCAGATGCA-3′, Rev 5′-AGCCTACTCATTGGGATCATCTTG-3′, ICAM-1 Fwd 5′-CACCCCAAGGACCCCAAGGAGAT-3′, Rev 5′-CGACGCCGCTCAGAAGAACCAC-3′; IL-10-Fwd 5′-AGTGAACTGCGCTGTCAATG-3′, Rev 5′-TTCAGGGTCAAGGCAAACTT-3′, and 18S-Fwd 5′-CGGCGACGACCCATTCGAAC-3′, Rev 5′-GAATCGAACCCTGATTCCCCGTC-3′.

### 4.6. ELISA

The levels of IL-6, CXCL-10, and Fg in the astrocyte conditioned medium were measured using sandwich ELISA kits. Mouse IL-6 (cat. # ab46100) and mouse fibrinogen SimpleStep (cat. #ab213478) ELISA kits were from Abcam (Cambridge, MA, USA), while CXCL-10 ELISA kit (cat. #BMS6018) was from Thermo Fisher Scientific.

### 4.7. ROS and NO Detection

Astrocyte ROS and NO production were detected using carboxy-H2DCFDA (Image-IT™ LIVE Green ROS Detection Kit, Invitrogen) and Griess assay (Promega), respectively, following manufacturers’ recommendations. To characterize time-dependence (kinetics) of Fg-induced ROS generation by astrocytes, cells were cultured in Nunc^TM^ 96 MicroWell^®^ plates. Fluorescence intensity was measured at excitation/emission maxima of 495/529 nm using Biotek Synergy H1 plate reader (BioTek Instruments Inc., Winooski, VT, USA).

### 4.8. Terminal Deoxynucleotidyl Transferase-dUTP Nick End Labeling (TUNEL) and LIVE/DEAD^®^ Viability/Cytotoxicity Assay

Click-iT^TM^ Plus TUNEL Assay for in situ apoptosis detection with Alexa Fluor^TM^ dye was purchased from Invitrogen and was performed following the manufacturers’ recommendations. Astrocytes were treated with either SFM alone, 4 mg/mL of Fg, 4 mg/mL of Fg in the presence of 22 μL of function-blocking antibody against ICAM-1, or 20 μL of PrP^C^ function-blocking peptide in SFM for 24 h. Then neurons on the bottom of the wells were used for TUNEL or LIVE/DEAD^®^ Viability/Cytotoxicity Assay (performed as recommended by the manufacturer).

### 4.9. Image Analysis

To detect proximity ligation of target proteins, cells were observed using a Keyence BZ-X710 Microscope with objectives 20× or 40×. Z-stacks of a selected constant-size area of interests (AOI) in each experimental well were acquired. The microscope settings were kept constant for all images to allow an adequate comparison. Acquired images were analyzed with ImageJ and the number of PLA-positive signals were counted using the Image-based Tool for Counting Nuclei plugin function in the ImageJ. The quantification of PLA signals per cell in 7 randomly selected, not overlapping AOIs with minimum of 3 cells were analyzed and averaged for each treatment group.

For detection of neuronal death with the TUNEL assay, images were taken using Olympus FV1200 (Tokyo, Japan) confocal microscope. For live/dead assay, images were taken using Olympus IX51 (Tokyo, Japan). Where appropriate, data were normalised per cell number in the selected AOI.

### 4.10. Statistical Analysis

The obtained data were analyzed using a Graph Pad Prism (San Diego, CA, USA). All data were expressed as mean ± SEM. The experimental groups were compared by one-way ANOVA. If ANOVA indicated a significant difference (*p* < 0.05), Bonferroni’s post hoc test was used to compare group means. ROS kinetic data were compared using a two-way ANOVA to compare the effect of two factors (time and treatment). Differences were considered significant if *p* < 0.05.

## Supplementary Materials

The following are available online at https://www.mdpi.com/1422-0067/22/5/2391/s1.

## Abbreviations

TBI	traumatic brain injury
STM	short-term memory
ICAM-1	intercellular adhesion molecule-1
PrP^C^	cellular prion protein
ROS	reactive oxygen species
NO	nitric oxide
Fg	fibrinogen
BBB	blood-brain barrier
AD	Alzheimer’s disease
PLA	proximity ligation assay
HFg	Hyperfibrinogenemia
DAPI	4′,6-diamidino-2-phenylindole
LPS	lipopolysaccharide
IL-10	interleukin 10
ELISA	enzyme-linked immunosorbent assay
TBHP	tert-butyl hydroperoxide
IFNγ	Interferon gamma
Aβ	amyloid-beta
NAPDH	Nicotinamide adenine dinucleotide phosphate
SFM	serum free medium
TUNEL	terminal deoxynucleotidyl transferase-dUTP nick end labeling
MAP2	Microtubule-associated protein 2
BSA	bovine serum albumin
