# Supplementary material for: Fibrinogen Interaction with Astrocyte ICAM-1 and PrPC Results in the Generation of ROS and Neuronal Death
Source: Int J Mol Sci. 2021 Feb 27;22(5):2391. doi: 10.3390/ijms22052391 (PMC7957521; doi:10.3390/ijms22052391)
Supplement: Supplementary file 1 [file ijms-22-02391-s001.pdf]

Supplementary Materials

1. Results and Discussion

1.1. Fibrinogen-induced activation of ICAM-1 on astrocytes

Data showed that mouse astrocytes expressed ICAM-1, and that Fg dose-dependently increased expression of this protein on the surface of astrocytes (Figure S1). Lipopolysaccharide (LPS) along with interferon gamma (IFNγ) (LPS+IFNγ) that were used as a positive control, increased the expression of astrocytic ICAM-1 compared to those in the control group, suggesting the validity of Fg effect (Figure S1). There was no expression of ICAM-1 when astrocytes were probed with IgG, confirming the specificity of the detecting antibody against ICAM-1 used in the study (Figure S1).

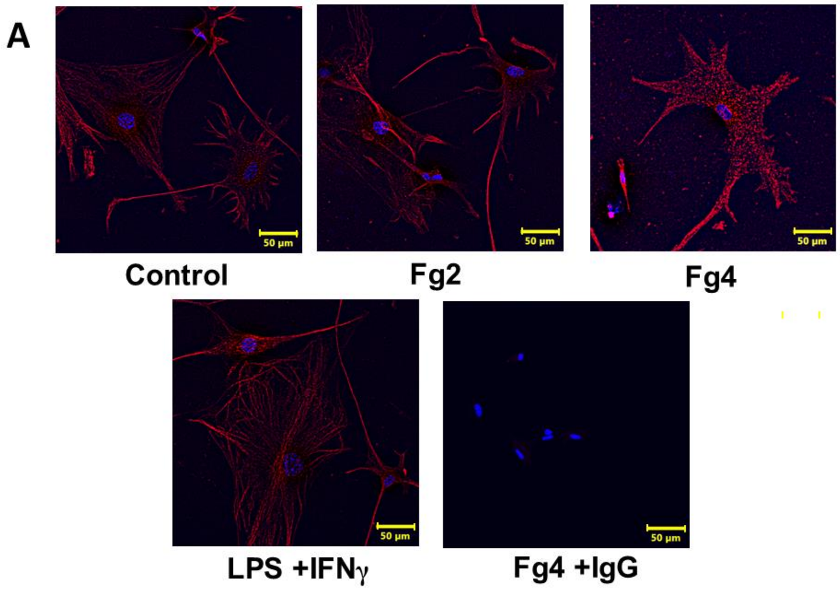

B

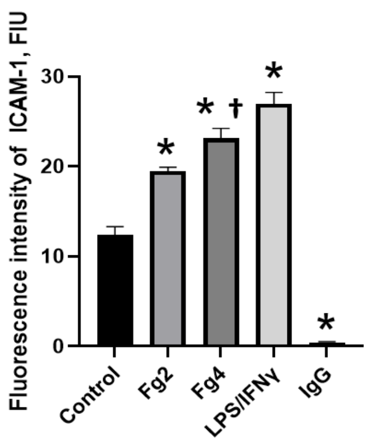

Figure S1. Fibrinogen-induced expression of intercellular adhesion molecule-1 (ICAM-1) on astrocytes. (a) Representative images of ICAM-1 (red) expression on the surface of mouse brain astrocytes treated with 2 mg/ml or 4 mg/ml of Fg (Fg2 and Fg4, respectively) or with medium alone (control). Treatment of cells with 1μg/ml of lipopolysaccharides along with 20ng/ml of interferon gamma (LPS + IFNγ) was used as a positive control. Cells were treated overnight. Cell nuclei were defined with 4′,6-Diamidine-2′-phenylindole dihydrochloride (blue). The detecting anti-ICAM-1 antibody that was used as a primary antibody was paired with a secondary antibody conjugated with Alexa-Fluor 594. One group of astrocytes treated with 4 mg/ml of Fg that were probed with IgG (Fg4+IgG), was used as a negative control group to define specificity of the ICAM-1 detecting antibody.; **(b)** A summary of the image analysis of the ICAM-1 expression on the surface of astrocytes presented as fluorescence intensity units (FIU). $P<0.05$ in all; ∗ - vs. Control, † - vs. Fg2; n=4

1.2. Fibrinogen-induced astrocyte death

Fibrinogen dose-dependently increased astrocyte death (Figure S2). These data indicate that Fg has toxic effects on astrocytes, which increase as the concentration of Fg approaches a level associated with hyperfibrinogenemia.

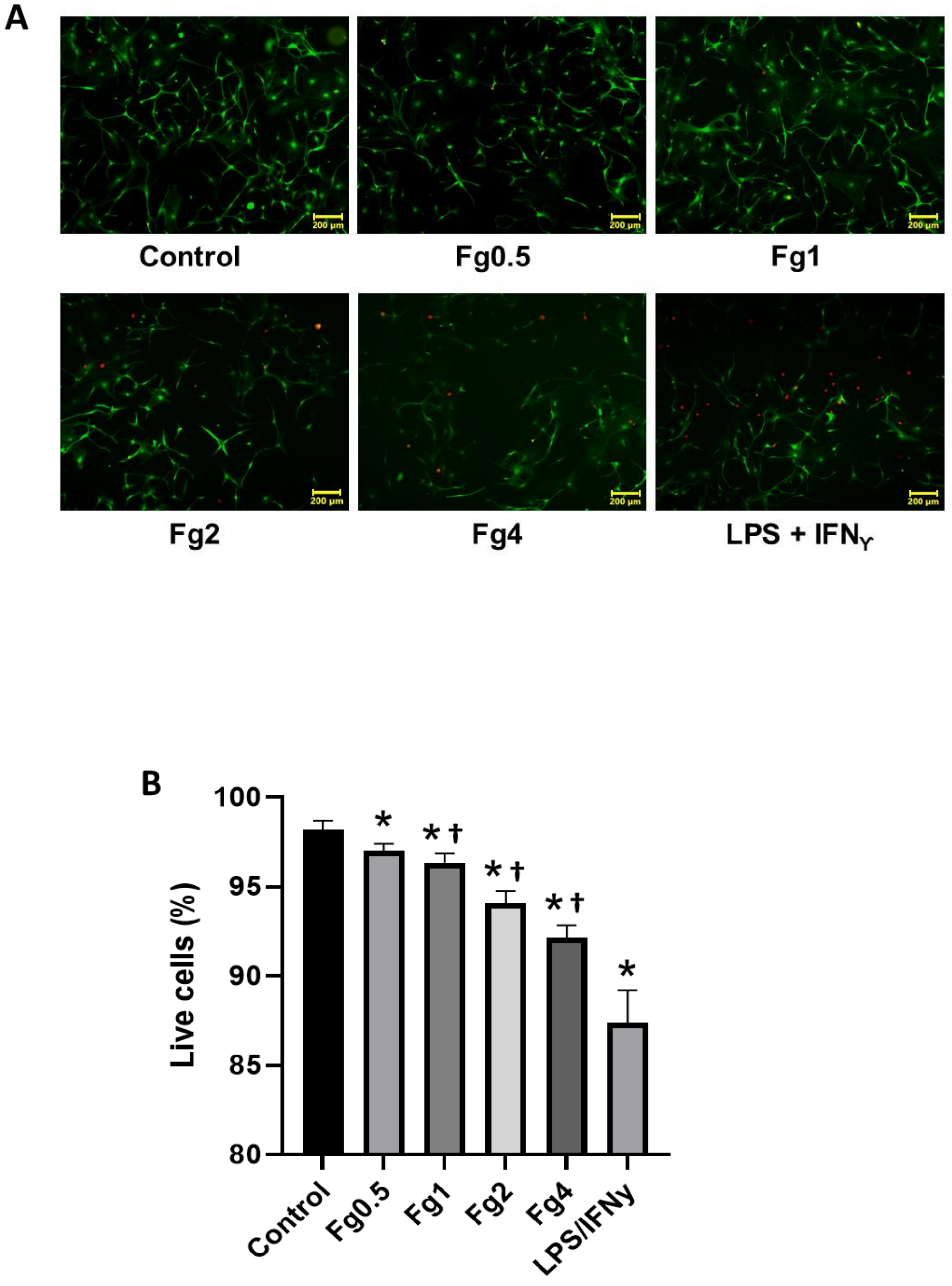

Figure S2. The effect of fibrinogen (Fg) on the viability of astrocytes shown by live/dead assay. (a) Representative images showing a fibrinogen (Fg)-induced increase in astrocyte death. Astrocytes were treated with Fg at 0.5, 1, 2, or 4 mg/ml (Fg0.5, Fg1, Fg2, or Fg4, respectively) overnight. Astrocytes in control group did not have Fg. The treatment of astrocytes with 1 μg/ml of lipopolysaccharide and 20 ng/ml of interferon gamma (LPS+IFNγ) overnight was used as a positive control. Live cells are visible in green, while dead cells are depicted with red.; **(b)** Summary of the image analyses for the detection of astrocyte cell death. The number of live cells was presented as a percent of a total number of cells. $P<0.05$ in all; ∗ - vs. Control, † - vs. lower concentration of Fg; n=4

1.3. *Interaction of fibrinogen with astrocytic ICAM-1 and PrP^C*

Use of negative controls resulted in negligible proximity ligation assay (PLA) signals, suggesting a high specificity of the used antibodies (Figure S3).

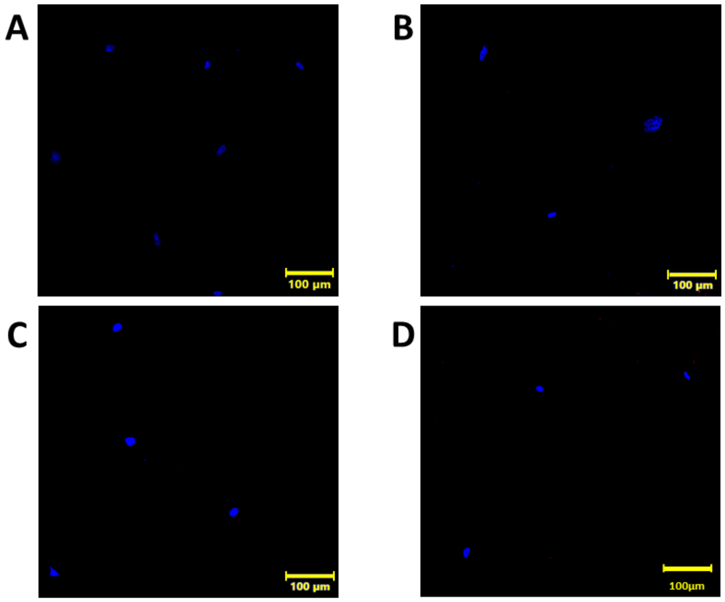

Figure S3. Interaction of fibrinogen (Fg) with its astrocyte receptors, intercellular adhesion molecule 1 (ICAM-1) and cellular prion protein (PrP^C) detected by proximity ligation assay (PLA), Negative controls. Representative images of *in situ* PLA signals (shown in red) depicting an interaction between Fg and astrocytic ICAM-1 or PrP^C for astrocytes treated with 1 mg/ml of Fg and only one of the two required antibodies: anti-Fg **(a)**, anti-ICAM-1 **(b)**, or anti-PrP^C **(c)**. In the fourth control **(d)** group, mouse IgG (Millipore, Cat. No. 12-371) as a serotype control for antibodies against ICAM-1 and PrP^C were used. Cellular nuclei were labeled with 4′,6-diamidino-2-phenylindole in blue**.**

2. Methods

2.1 *Immunocytochemistry*

Confluent astrocytes were treated with Fg (2 or 4 mg/ml) or with LPS co-stimulated with IFNγ overnight at 37°C. After incubation cells were washed with phosphate buffered saline (PBS) and fixed with 4% paraformaldehyde. The fixed astrocytes were treated with a permeating solution containing 0.1% Triton™ X-100 for 10 minutes before blocking with 3% bovine serum albumin in PBS for 1 hour. Astrocytes were incubated with an antibody against the ICAM-1 primary antibody from Biolegend (Cat. No. 116102) (1:200) and kept in the dark at 4°C overnight. LEAF™ Purified Rat IgG2b, ƙ isotype control from Biolegend (Cat. No. 400622) (1:200) was used as an isotype control for ICAM-1. Cells were incubated for 2 hours at 1:200 dilution of an appropriate secondary antibody that was conjugated with Alexa Fluor-

594. Cells were washed with PBS between the incubations. Mounting media with 4′,6-diamidino-2-phenylindole (DAPI) was used to label cell nuclei.

To detect ICAM-1 expression on astrocytes, immunofluorescently-stained astrocytes were observed using Olympus FV1000 (Tokyo, Japan) laser-scanning confocal microscope and the obtained images were deconvoluted using 2D deconvolution algorithm of the CellSens Dimension 1.11 software (Tokyo, Japan). Regions of interest (ROI) of equal size were selected in each image where astrocytes expressing ICAM-1 were observed. Mean fluorescence intensity of the ICAM-1 signal was measured and normalized to number of cells, defined by DAPI in each ROI. Then data were averaged for each experimental group.
